# Plasma C-Type Natriuretic Peptide as a Predictor for Therapeutic Response to Metoprolol in Children with Postural Tachycardia Syndrome

**DOI:** 10.1371/journal.pone.0121913

**Published:** 2015-03-26

**Authors:** Jing Lin, Zhenhui Han, Hongxia Li, Selena Ying Chen, Xueying Li, Ping Liu, Yuli Wang, Chaoshu Tang, Junbao Du, Hongfang Jin

**Affiliations:** 1 Department of Pediatrics, Peking University First Hospital, Beijing, China; 2 Department of Pediatrics, Kaifeng Children’s Hospital, Henan, China; 3 University of California San Diego, La Jolla, California, United States of America; 4 Department of Medical Statistics, Peking University First Hospital, Beijing, China; 5 Department of Physiology and Pathophysiology, Peking University Health Science Center, Beijing, China; 6 Key Laboratory of Molecular Cardiology, Ministry of Education, Beijing, China; University of Pennsylvania Perelman School of Medicine, UNITED STATES

## Abstract

POTS is a global public-health disease, but predictor for therapeutic response to metoprolol in children with POTS is lacking. This study was designed to investigate predictive value of plasma C-type natriuretic peptide (CNP) in the therapeutic efficacy of metoprolol on postural tachycardia syndrome (POTS) in children. Totally 34 children with POTS and 27 healthy children were included in the study. The head-up test or head-up tilt test was used to check heart rate and blood pressure from supine to upright in subjects. A double antibody (competitive) sandwich immunoluminometric assay was used to detect plasma CNP. Metoprolol was used to treat children with POTS. The difference in plasma concentrations of CNP between responders and non-responders was compared. An ROC curve was used to analyze plasma CNP to predict efficacy of metoprolol on POTS in children. Plasma CNP in children with POTS was significantly higher than that of healthy children [(51.9 ± 31.4) vs. (25.1 ± 19.1) pg/ml, *P* <0.001]. Plasma CNP in responders to metoprolol was significantly higher than non-responders [(59.1 ± 33.5) vs. (34.8 ± 16.7) pg/ml, *P* = 0.037] before treatment. The ROC curve showed that area under the curve was 0.821 (95% CI 0.642–0.999). The cut-off value of plasma CNP > 32.55 pg/ml yielded a sensitivity of 95.8% and specificity of 70% in predicting therapeutic efficacy of metoprolol on POTS children. Plasma CNP might serve as a useful predictor for the therapeutic efficacy of metoprolol on POTS in children.

## Introduction

Postural tachycardia syndrome (POTS) is a subtype of orthostatic intolerance (OI). Clinically, for children and adolescents, POTS patients have a heart rate ≥ 40 beats/min or a maximum heart rate ≥ 120 beats/min during the head-up test or head-up tilt test, and are clinically accompanied by dizziness or vertigo, chest tightness, headache, palpitations, paleness, blurred vision, fatigue, morning discomfort, and even syncope. All the symptoms last no less than 1 month. Some POTS children have more severe clinical symptoms which impact their studies and daily life [1,2]. Therefore, timely and effective treatment is particularly important. The β-blocker, metoprolol, plays an important role in the treatment of POTS largely by blocking β-adrenoceptor, and as a result, blocking the action of high level of catecholamines in the blood stream [3]. However, Chen et al. found that after 3–6 months of treatment, the efficacy of metoprolol on POTS was only 57.89% [4]. Therefore, looking for useful biomarkers to predict whether the patient is suitable for metoprolol therapy would be of great significance.

C-type natriuretic peptide (CNP) is a small molecule protein that was isolated by Sudoh et al. from the tissue of a porcine brain in 1990 [5]. It was the third natriuretic peptide, after the atrial natriuretic peptide (ANP) and the brain natriuretic peptide (BNP). The original human CNP precursor is composed of 126 amino acids. After the removal of the signal peptide which is composed of 23 amino acids at the amino-terminal, the original CNP is generated composing 103 amino acids CNP22 and CNP53 [6]. CNP22 was generally considered an active form and CNP53 an intermediate storage [7]. Experiment *in vitro* indicated that CNP played a role in the reduction of blood pressure and the inhibition of smooth muscle cell proliferation, natriuresis, diuresis, vasodilation and inhibition of the renin- angiotensin system [8]. It was also involved in the pathogenesis of heart failure [9–13] and myocardial infarction [14]. More interestingly, CNP in plasma could also be used as a biomarker of bone injury induced by steroid hormones [15]. Based on its features of easy detection, stable content in circulation, and convenience of saving, numerous studies used CNP as a clinical biomarker. Importantly, Takekoshi et al. found that CNP, ANP and BNP could increase the catecholamine synthesis by increasing the expression of tyrosine hydroxylase mRNA via the cGMP / PKG pathway [16]. Springer et al. found that CNP could activate the guanylate cyclase receptor- related CNP receptor (NPR-A and NPR-B) and increase the heart rate by restraining the activity of phosphodiesterase (PDE3) [17]. The increment of catecholamines in plasma is an important pathophysiological mechanism for POTS [18, 19], and it can worsen the symotoms of POTS. So, we assumed that plasma CNP could likely reflect the level the catecholamine in the blood and reflect the heart rate to a certain extent. Therefore, the present study was undertaken to examine if the plasma level of CNP could predict the efficacy of metoprolol in the treatment of children with POTS.

## Materials and Methods

### Subjects

Sixty-one subjects were recruited in the study, including 34 children with POTS from the outpatient and inpatient pediatric department of Peking University First Hospital [aged 7–16 (11.7 ± 2.0) years, 18 males and 16 females] with manifestations of dizziness or vertigo, chest tightness, headache, palpitations, paleness, blurred vision, fatigue, morning discomfort, or syncope, diagnosed as POTS [1]. All children fulfilled a complete history taking and laboratory investigations including ECG, EEG, blood glucose and blood biochemistry tests, and a cranial CT or MRI, to exclude a transient loss of consciousness caused by cardiac, neurologic, metabolic and psychogenic diseases. Twenty-seven healthy children, aged 9–14 (11.4 ± 1.7) years, served as controls, based on the normal findings of medical history taking, physical examination, and ECG, etc. All children were told the purposes of the research and agreed to provide relevant research information. The study was approved by the Peking University First Hospital Ethics Committee, China. Written informed consent was obtained from all study subjects/parents, the next of kin, or guardians of the minors/children enrolled in our study.

### Head-up test and head-up tilt test

#### Head-up test (HUT)

Children were asked to stop using all drugs that might affect autonomic function before the test. The test was performed in a quiet room at suitable room temperature equipped with medical resuscitation facilities. The patients were required to lie down for at least ten minutes. The heart rate and blood pressure were monitored by a Dash 2000 Multi-Lead Physiological Monitor (General Electric, NY, New York, USA). The children were then asked to stand up for ten minutes, with heart rate and blood pressure being monitored. If a positive response appeared within 10 minutes of the upright position, the test would be discontinued. If changes in the heart rate and blood pressure were within normal range during HUT, subjects would undertake the HUTT.

#### Head-up tilt test (HUTT)

All children were fasted before the test, and stopped using all drugs that might affect autonomic function. Children lay on the tilt table (HUT-821; Beijing Juchi, Beijing, China) as their heart rate and blood pressure were consistently monitored by a Dash 2000 Multi-Lead Physiological Monitor until the heart rate stabilized. The table was then tilted to a 60° angle, and the heart rate and blood pressure were monitored until either a positive response appeared or the test (with a process of 45 minutes) was complete. During the test, if an increase in heart rate ≥ 40 beats/min or the maximum heart rate ≥ 120 beats/min was accompanied by two of the following symptoms such as dizziness or vertigo, chest tightness, headache, palpitations, a change in paleness, blurred vision, fatigue, morning discomfort, or syncope during tilt, the diagnosis of POTS was made[20].

### POTS diagnostic criteria

POTS diagnostic criteria [20,21]: (1) normal heart rate when supine and no evidence of any cardiovascular diseases; (2) ≧2 of dizziness, headache, fatigue, blurred vision, chest tightness, palpitations, hand tremors, or syncope after standing; (3) increment in heart rate ≥ 40 beatsmin^-1^ or maximum heart rate ≥ 120 beats min^-1^ after standing during the head-up test or head-up tilt test, with at least 2 of the following symptoms: dizziness or vertigo, lightheadedness, headache, palpitations, complexion change, blurred vision, fatigue, discomfort in the morning, and syncope for severe cases; (4) symptoms relieved or diminished by recumbence and symptoms for ≧1 month; and (5) exclusion of other cardiac, neurologic or metabolic diseases.

### Efficacy evaluation and follow-up

All patients were evaluated by using symptom scoring and the head-up test or head-up tilt test as a basal investigation before treatment. All patients were treated with metoprolol (12.5 mg, twice/day) for 3 months. The symptoms were evaluated during follow-up for each child after 3 months of treatment in the outpatient department or over telephone. The severity of the symptoms of the disease was calculated by using symptom score system [22, 23] and the adverse effects of patients were recorded. As for symptom score system, ten of the main clinical symptoms are syncope, dizziness, lightheadedness, nausea, heart palpitations, headaches, hand tremors, sweating, blurred vision and inattention [22]. 0 point was given for the absence of POTS symptoms; 1 point was given for one symptom once a month; 2 points were given for one symptom 2–4 times per month; 3 points were given for one symptom 2–7 times per week; and 4 points were given for one symptom at least once per day. Symptom scoring was repeated to evaluate the overall severity of the disease at the end of the treatment. The patients were considered as responders if the decrease in symptom score was ≥ 2 points after treatment. They were considered as non-responders if the decrease in symptom score was < 2 points after treatment [23,24]. Twenty-three patients repeated the head-up test to show the heart rate increment after treatment for 3 months.

### Determination of plasma CNP

POTS patients and healthy children were fasted before blood sampling. Four ml of blood samples were drawn at 8:00 in the morning in supine position. and were saved within an EDTA anticoagulant tube. Plasma was obtained after centrifuging at 2000 g for 20 minutes at 4°C. Aprotinin was added into the plasma and the plasma samples were stored in a -80°C refrigerator. The plasma CNP was measured in a sandwich immunoluminometric assay (Phoenix Pharmaceuticals, Burlingame, California, USA). The assay employed 2 polyclonal rabbit antibodies to the CNP, and had an analytical detection limit of 10 pg/ml.

### Statistical analysis

The data were analyzed with the SPSS 17.0 software. Enumeration data are described in the form of case number, and measurement data are described in the form of mean ± *s*. A χ^2^ test and *t* test were used for the analysis of enumeration data and measurement data, respectively, between the two groups. A paired *t* test was applied for the analysis of measurement data of one patient before and after treatment. The ROC curve was used to evaluate the predictive value of CNP in assessing the therapeutic effect of metoprolol. The area under curve (AUC) was used to indicate the predictive value. An AUC from 0.5 to 0.7 indicates a lower predictive value; AUC from 0.7 to 0.9 indicates a moderate predictive value; AUC > 0.9 indicates a high predictive value. *P* value < 0.05 was considered statistically significant.

## Results

### Demographic and hemodynamic parameters and plasma CNP between patients with POTS and healthy children

There was no statistical difference between the two groups in age, height, weight, supine systolic blood pressure, supine heart rate, or systolic and diastolic blood pressure after standing (*P* > 0.05). Compared to healthy children, the supine diastolic blood pressure was low in POTS patients [(61 ± 7) vs. (68 ± 8) mmHg, *t* = 3.494, *P* < 0.001], but the upright maximum heart rate was significantly increased in POTS patients [(119 ± 14) vs. (105 ± 11) beats/min, *t* = -4.242, *P* < 0.001] and the increment in heart rate was significantly obvious in POTS patients after standing [(39 ± 12) vs. (22 ± 7)/min, *t* = -6.951, *P* < 0.001]. The plasma concentrations of CNP were significantly increased in POTS patients [(51.9 ± 31.4) vs. (25.1 ± 19.1) pg/ml, *t* = -3.908, *P* < 0.001]. (Table 1)

**Table 1:** Baseline characteristics of study participants.

Items	Control group	POTS group	***t/***χ^2^	***P***
**Cases**	27	34		
**Sex**	12/15	18/16	0.435	0.51
**Age, yrs**	11.4 ± 1.7	11.7 ± 2.0	-0.488	0.627
**Height, cm**	151.7 ± 11.4	153.8 ± 11.0	-0.723	0.473
**Weight, kg**	42.6 ± 10	46.7 ± 6.1	-1.874	0.068
**Supine SBP, mmHg**	110 ± 12	106 ± 8	1.566	0.127
**Supine DBP, mmHg**	68 ± 8	61 ± 7	3.494	0.001
**Supine HR, mmHg**	83 ± 11	80 ± 9	1.357	0.18
**Upright SBP, mmHg**	113 ± 11	113 ± 10	-0.164	0.871
**Upright DBP, mmHg**	70 ± 7	71 ± 8	-0.386	0.701
**Upright HR, beats/min**	105 ± 11	119 ± 14	-4.242	<0.001
**Delta HR, beats/min**	22 ± 7	39 ± 12	-6.951	<0.001
**CNP (pg/ml)**	25.1 ± 19.1	51.9 ± 31.4	-3.908	<0.001

POTS: Postural tachycardia syndrome; SBP:systolic blood pressure;DBP: diastolic blood pressure;HR: heart rate; delta HR:upright heart rate minus supine heart rate; CNP: C-type natriuretic peptide.

### Symptom scores and heart rate changes of patients with POTS after metoprolol treatment

**Fig 1:**
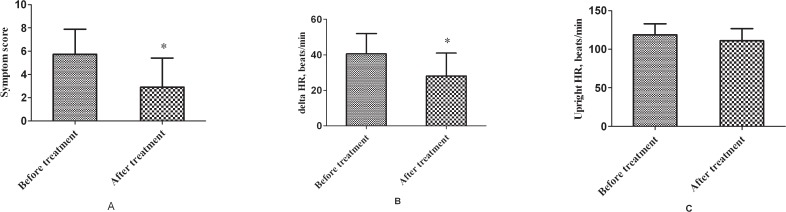
Symptom scores and heart rate changes of patients with POTS after metoprolol treatment. Compared to the symptom scores before treatment, the scores were significantly decreased after treatment in 34 children with POTS [(5.7 ± 2.2) vs. (2.9 ± 2.5), *t* = 9.232, *P* <0.001)]. Twenty-three patients repeated the head-up test, showing that the increment in heart rate induced by postural changes was significantly reduced after treatment [(41 ± 11) vs. (28 ± 13) beats/min, *t* = 3.476, *P* = 0.002]. * *P* < 0.01 compared with before treatment.

Compared to the symptom scores before treatment, the scores were significantly decreased after treatment in 34 children with POTS [(5.7 ± 2.2) vs. (2.9 ± 2.5), *t* = 9.232, *P* <0.001)]. Twenty-three patients repeated the head-up test, showing that the increment in heart rate induced by postural changes was significantly reduced after treatment [(41 ± 11) vs. (28 ± 13) beats/min, *t* = 3.476, *P* = 0.002]. (Fig. 1)

### Plasma concentration of CNP between responders and non-responders to metoprolol

The concentration of plasma CNP was significantly different between the responders and non-responders before treatment (*P* < 0.05). In responders, the plasma concentration of CNP was significantly higher than that of the non-responders [(59.1 ± 33.5) vs. (34.8 ± 16.7) pg/ml, *t* = -2.177, *P* < 0.05]. (Table 2)

**Table 2:** Comparisons of baseline heart rate increments, symptom scores and plasma CNP level between responders and non-responders to metoprolol in POTS children

Patients	n	Delta HR, beats/min	Symptom scores	Plasma CNP (pg/ml)
**Responders**	24	38±13	5.9±2.2	59.1±33.5
**Non-responders**	10	42±9	5.4±2.2	34.8±16.7
***t***		0.913	-0.581	-2.177
***P***		0.368	0.565	0.037

CNP: C-type natriuretic peptide.

### Symptom outcome among patients responded to metoprolol

Twenty-four patients claimed for dizziness and lightheadedness, 20 of them were improved, 2 got worsened and 2 unchanged. Seventeen patients claimed for headaches, 13 of them were improved, 1 got worsened and 3 patients unchanged. Fourteen patients claimed for inattention, 9 of them were improved, 1 got worsened and 4 patients remained unchanged. Eight patients claimed for heart palpitations, 4 of them were improved, 2 got worsened and 2 unchanged. Six patients claimed for nausea, syncope and sweating, 5 of them were improved and 1 patient remained unchanged. Five patients claimed for blurred vision, 4 were improved but 1 unchanged. At last, 4 patients claimed for hand tremors and all of them were improved. (Table 3)

**Table 3:** Changes in symptoms among the patients responded to metoprolol.

Symptoms	Cases	Improved	Unchanged	Worsened
**dizziness**	24	20	2	2
**lightheadedness**	24	20	2	2
**headaches**	17	13	3	1
**inattention**	14	9	4	1
**heart palpitations**	8	4	2	2
**nausea**	6	5	1	0
**syncope**	6	5	1	0
**sweating**	6	5	1	0
**blurred vision**	5	4	1	0
**hand tremors**	4	4	0	0

### Severity of the symptoms, increment in heart rate from supine to upright and the maximum upright heart rate were positively correlated with plasma CNP

**Fig 2:**
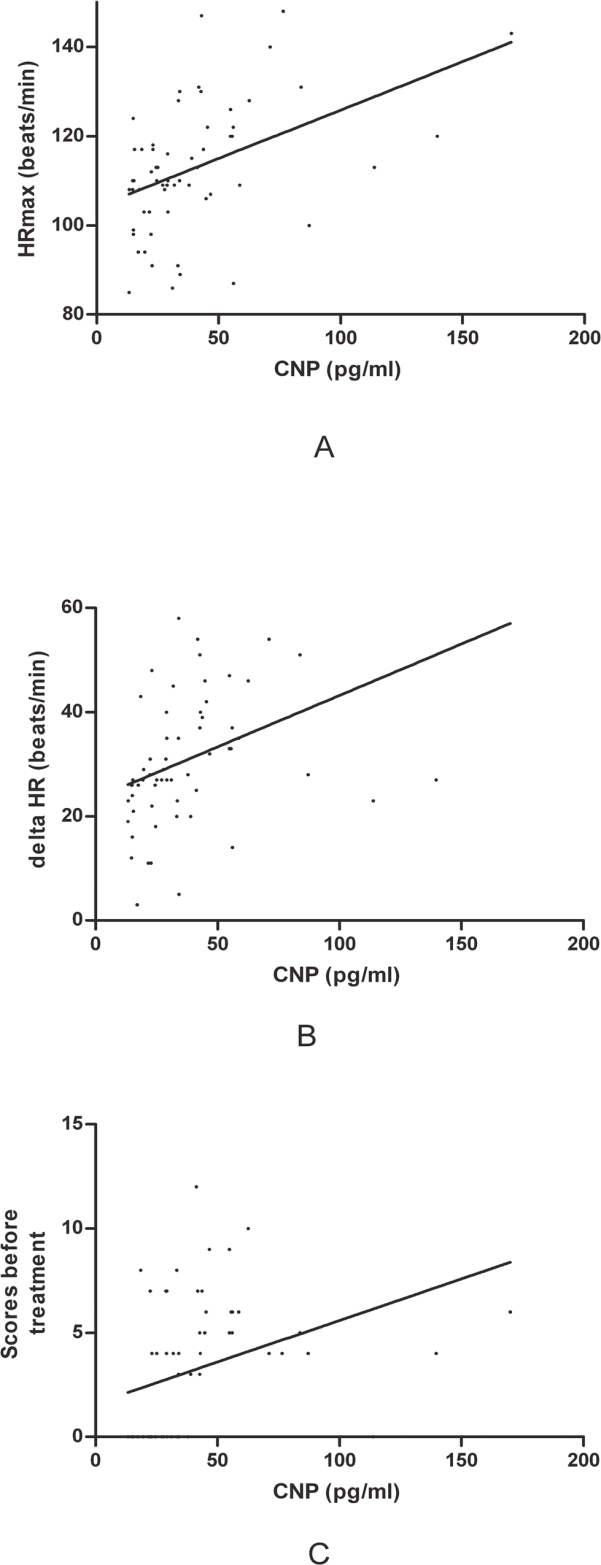
Linear correlation of plasma CNP with the upright maximum heart rate, increased heart rate from supine to upright and symptom scores. A: concentration of CNP was positively correlated with the upright maximum heart rate. Correlation coefficient r = 0.445, r^2^ = 0.20, (*P* < 0.001); B: concentration of plasma CNP was positively associated with increased value of heart rate from supine to upright. Correlation coefficient r = 0.434, r^2^ = 0.19, (*P* < 0.001); C: concentration of plasma CNP was positively correlated with symptom scores. Correlation coefficient r = 0.361, r^2^ = 0.13, (*P* < 0.01)

The symptom scores, the increment in heart rate from supine to upright position and the maximum upright heart rate were found to positively correlate with plasma concentrations of CNP (symptom scores: *r* = 0.361, r^2^ = 0.13, *P* = 0.004; increment in heart rate: *r* = 0.434, r^2^ = 0.19, *P* < 0.001; and maximum upright heart rate: *r* = 0.445, r^2^ = 0.20, *P* < 0.001, respectively). (Fig. 2)

### The predictive value of plasma CNP for therapeutic efficacy of metoprolol by the ROC curve

**Fig 3:**
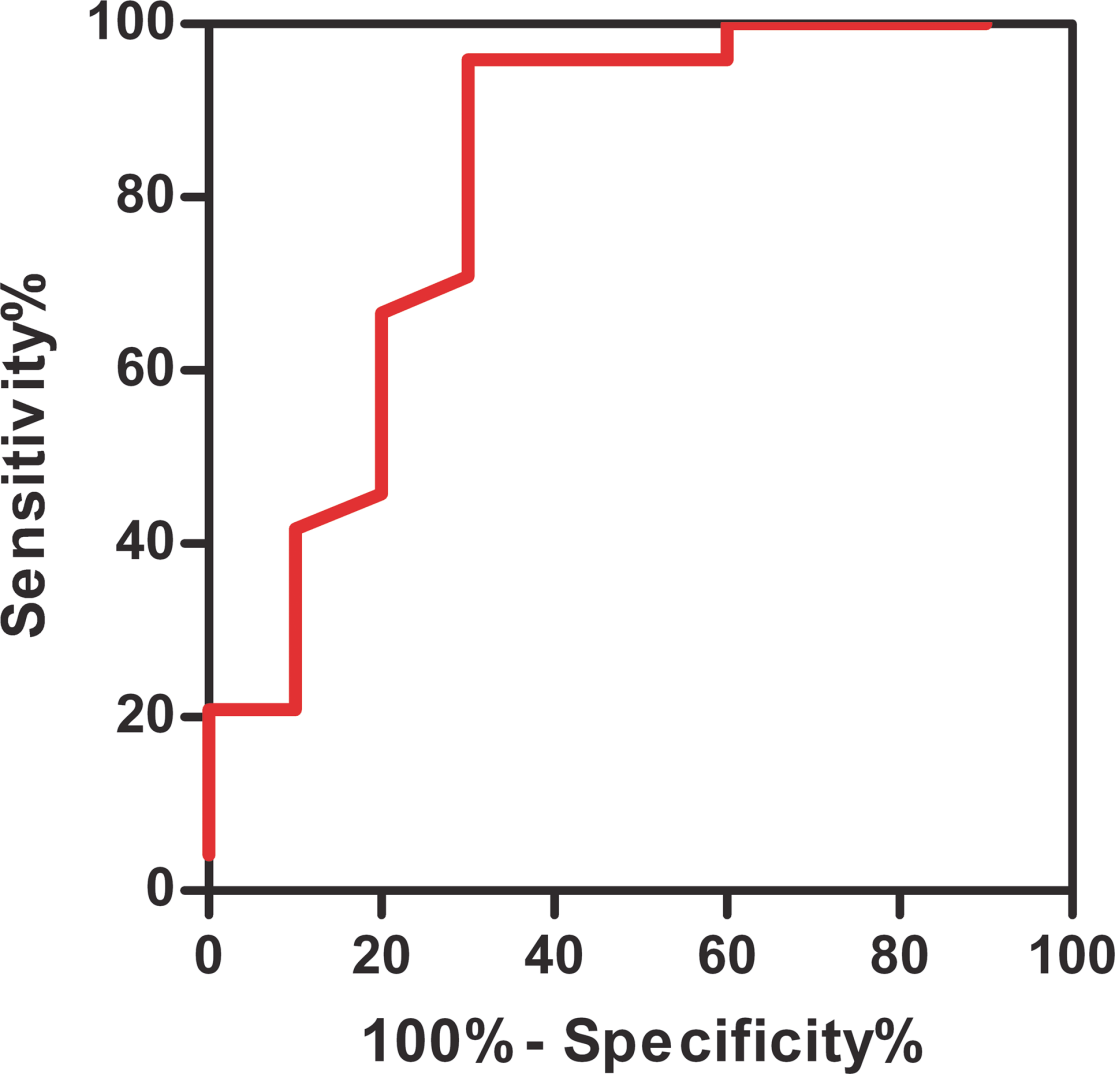
The receiver-operating characteristic (ROC) curve for plasma CNP levels for predicting the therapeutic effect of metoprolol. The y-axis represents the sensitivity to predict the effectiveness of different plasma CNP levels in metoprolol therapy. The x-axis represents the false-positive rate (1-specificity) of the prediction. When the area under the curve was 0.5 to 0.7, the prediction value was low; when it was 0.7 to 0.9, the prediction value was medium; and when it was > 0.9, the diagnostic value was relatively high. The area under the curve was 0.821 (*P* < 0.01).

The ROC curve showed that the area under the curve was 0.821, (95% CI 0.642–0.999), indicating that it yielded a high predictive value for using a pre-treatment plasma concentration of CNP to predict the efficacy of oral metoprolol therapy in patients with POTS. When plasma concentration of CNP was > 32.55 pg/ml, the sensitivity and specificity for predicting metoprolol efficacy were 95.8% and 70%, respectively. (Fig. 3)

## Discussion

Low et al. reported that the incidence of POTS in the adult population was 170/100,000 [25] and another study showed that the prevalence of POTS in young males was around 10% [26]. Children with POTS often have headache, dizziness, palpitations, blurred vision and other symptoms, but also a poor quality of sleep, bladder dysfunction, nausea, abdominal pain, diarrhea, constipation and other symptoms. Syncope and other serious clinical manifestations can hinder patients’ daily life and school performance, potentially causing a greater negative impact on their psychological state. Therefore, timely and standardized treatment is very important.

Metoprolol is one of the main drugs for POTS currently. It is used to treat POTS by blocking β-adrenoceptor, and as a result, blocking high levels of catecholamines in circulation, and lowering the heart rate [3]. Previous studies showed that the efficacy of metoprolol on POTS was variable. Zhang et al. showed that metoprolol had a relatively high efficacy in the therapy of POTS [27], but Chen et al. indicated that the effective rate was only 57.89% [4]. Therefore, if the efficacy of metoprolol had been predicted before treatment, it would have been possible to significantly improve the treatment efficacy of metoprolol. In patients with POTS, during the position changes from supine to upright, such stimuli would result in excessively reduced venous return and central blood volume causing an increase in the arginine vasopressin (AVP) level as well as a proportional high level of catecholamine which would cause POTS symptoms. Coppeptin, a joint glycopeptide of AVP, could be released equally with AVP, and they remain at stable levels in the blood[20]. Therefore, Zhao et al. attempted to predict the therapy effectiveness of metoprolol for children with POTS by using plasma copeptin. The results revealed that when the concentration of plasma copeptin was 10.2 pmol/L before treatment, the sensitivity and specificity of predicting metoprolol efficacy were 90.5% and 78.6%, respectively [20]. However, the plasma copeptin in the study is detected by enzyme-linked immunosorbent assay (ELISA), a method with relatively complex operating procedure. Zhang Q et al. attempted to predict the therapeutic effectiveness of metoprolol for children with POTS by using plasma norepinephine. The results revealed that when the concentration of plasma norepinephine was 3.59 pg/ml before treatment, the sensitivity and specificity of predicting metoprolol efficacy were 76.9% and 91.7%, respectively [28]. But the plasma norepinephine was very unstable in circulation.

A previous study revealed that plasma CNP played a role in increasing the secretion of plasma catecholamine and accelerating the heart rate [16,17]. The increased plasma level of catecholamine was suggested to be involved in the pathogenesis of POTS [18,19], evidenced by a heart rate increment in such children. Therefore, plasma CNP might be reflective to some extents to the clinical manifestations of children with POTS. Furthermore, at present, many small peptide molecules including CNP can be identified conveniently by an immunoluminometric assay.

As was expected, in this study the concentration of plasma CNP in children with POTS was significantly higher than in healthy children. Plasma CNP levels in children presented a positive correlation with clinical severity (symptom score, increment in heart rate from supine to upright position and the maximum heart rate after standing). More importantly, for responders to metoprolol, their plasma concentrations of CNP before treatment were significantly higher than those of non-responders. ROC curve analysis showed that plasma CNP could be a predictive biomarker for the efficacy of metoprolol in the treatment of POTS patients.

There are two reasons for the increased plasma catecholamine levels and heart rate in children with POTS. First, during upright, about 500–1000 ml of blood moves from the upper part body to the lower part, primarily into the leg and abdomen. The venous filling is reduced and accordingly blood return to the heart is reduced, resulting in a drop of cardiac output and blood pressure. When the cardiac baroreceptors senses the signal of a drop in blood pressure, sympathetic nerve efferent activity increases, inducing a greater vascular resistance. At the same time, the heart rate accelerates as a compensatory means for cardiac output [29,18]. During the pathophysiological processes, there is an increment in plasma norepinephrine due to the excessive activation of the sympathetic system. Compared to supine position, the plasma levels of norepinephrine raised 2–3 times after standing in a normal person [18]. A further study showed that there was an increased plasma concentration of norepinephrine in patients with neurally-mediated syncope (NMS) by the head-up tilt test. Compared to the normal control group, the norepinephrine concentrations in patients with NMS were greatly increased during syncopal episodes [19]. Second, the plasma CNP could increase catecholamine synthesis by increasing the mRNA expression of tyrosine hydroxylase via the cGMP/PKG pathway [16]. Plasma CNP could also activate the guanylate cyclase related CNP-receptor (NPR-A and NPR-B) and restrain the activity of phosphodiesterase (PDE3) to increase the heart rate [17]. Therefore, children with POTS congenitally present a high concentration of CNP, and when there is a central hypovolemia, it might aggravate the pathophysiologic process. Finally, it was confirmed by Liao et al. that there was peripheral vasodilation in children with POTS. They explored the vascular endothelial function in children with POTS through the application of color Doppler ultrasound to detect the flow-mediated vasodilation response (FMD). They found that the basal FMD in children with POTS was significantly higher than in the control group (11 ± 3% vs. 6 ± 2%, *p* <0.001) [30]. Therefore, CNP in children with POTS assists in dilating blood vessels, speeding up the heart rate and increasing the concentration of catecholamine in the circulatory system.

The main mechanism for metoprolol in the treatment of POTS is that it blocks β-adrenoceptor, slows down the heart rate, and reduces the high levels of circulating catecholamines. Plasma CNP can reflect the severity of the pathophysiology of a patient with POTS. According to this study, when the plasma concentration of CNP is up to 32.55 pg/ml in a patient with POTS, metoprolol can possibly improve the clinical symptoms of pediatric POTS.

There are limitations of this study, however. It is a single-centered study and enlargement of the case number is in need in the future. Anyway, the present study discovered for the first time the differences in the plasma concentration of CNP between patients with POTS and healthy children. Of great significance are the findings that plasma concentrations of CNP could predict the efficacy of metoprolol in treating POTS, which provided an important stepping stone for the individualized treatment of POTS.

## References

[1] Freeman R, Wieling W, Axelrod FB, Benditt DG, Benarroch E, Biaggioni I, et al Consensus statement on the definition of orthostatic hypotension, neurally mediated syncope and the postural tachycardia syndrome. Clin Auton Res.2011; 21: 69–72. 10.1007/s10286-011-0119-5 21431947

[2] Johnson JN, Mack KJ, Kuntz NL, Brands CK, Porter CJ, Fischer PR.. Postural orthostatic tachycardia syndrome: a clinical review. Pediatr Neurol. 2010;42:77–85. 10.1016/j.pediatrneurol.2009.07.002 20117742

[3] Lai CC, Fischer PR, Brands CK, Fisher JL, Porter CB, Driscoll SW, et al Outcomes in adolescents with postural orthostatic tachycardia syndrome treated with midodrine and beta-blockers. Pacing Clin Electrophysiol.2009; 32: 234–238. 10.1111/j.1540-8159.2008.02207.x 19170913

[4] Chen L, Wang L, Sun J, Qin J, Tang C, Jin H, et al Midodrine hydrochloride is effective in the treatment of children with postural orthostatic tachycardia syndrome. Circ J. 2011;75: 927–931. 21301135 10.1253/circj.cj-10-0514

[5] Sudoh T, Minamino N, Kangawa K, Matsuo H. C-type natriuretic peptide (CNP): a new member of natriuretic peptide family identified in porcine brain. Biochem Biophys Res Commun. 1990;168: 863–870. 2139780 10.1016/0006-291x(90)92401-k

[6] Barr CS, Rhodes P, Struthers AD. C-type natriuretic peptide. Peptides. 1996;17:1243–1251. 8959763 10.1016/s0196-9781(96)00110-6

[7] Potter LR, Yoder AR, Flora DR, Antos LK, Dickey DM. Natriuretic peptides: their structures, receptors, physiologic functions and therapeutic applications. Handb Exp Pharmacol. 2009;191: 341–366. 10.1007/978-3-540-68964-5_15 19089336 PMC4855512

[8] Takida S, Elm quist BJ, Trachte GJ. C-type natriuretic peptide attenuates evoked dopamin efflux by influencing Ggoalpha. Hypertention. 1999;33: 124–129. 9931092 10.1161/01.hyp.33.1.124

[9] Del Ry S, Maltinti M, Emdin M, Passino C, Catapano G, Giannessi D. Radioimmunoassay for plasma C-type natriuretic peptide determination: a methodological evaluation. Clin Chem Lab Med. 2005; 43: 641–645. 16006261 10.1515/CCLM.2005.110

[10] Del Ry S, Passino C, Maltinti M, Emdin M, Giannessi D. C-type natriuretic peptide plasma levels increase in patients with congestive heart failure as a function of clinical severity. Eur J Heart Failure. 2005, 7: 1145–1148. 15922659 10.1016/j.ejheart.2004.12.009

[11] Del Ry S, Maltinti M, Piacenti M, Passino C, Emdin M, Giannessi D. Cardiac production of C-type natriuretic peptide in heart failure. J Cardiovasc Med. 2006; 7: 397–399. 16721200 10.2459/01.JCM.0000228688.94709.5a

[12] Del Ry S, Giannessi D, Maltinti M, Prontera C, Iervasi A, Colotti C, et al Increased levels of C-type natriuretic peptide in patients with idiopathic left ventricular dysfunction. Peptides. 2007; 28: 1068–1073. 17428580 10.1016/j.peptides.2007.03.002

[13] Del Ry S, Maltinti M, Cabiati M, Emdin M, Giannessi D, Morales MA. C-type natriuretic peptide and its relation to non invasive indices of left ventricular function in patients with chronic heart failure. Peptides. 2008; 29: 79–82. 18063200 10.1016/j.peptides.2007.10.022

[14] Qvigstad E, Moltzau LR, Aronsen JM, Nguyen CH, Hougen K, Sjaastad I, et al Natriuretic peptides increase beta1-adrenoceptor signaling in failing hearts through phosphodiesterase 3 inhibition. Cardiovasc Res. 2010; 85: 763–772. 10.1093/cvr/cvp364 19900965

[15] Prickett TC, Lyver A, Wilson R, Espiner EA, Sullivan MJ. C-type natriuretic peptide:a novel biomarker of steroid induced bone toxicity in children with acute lymphoblasticleukemia (ALL). Peptides. 2012; 36:54–59. 10.1016/j.peptides.2012.04.017 22564489

[16] Takekoshi K, Ishii K, Isobe K, Nomura F, Nammoku T, Nakai T. Effects of natriuretic peptides (ANP, BNP, CNP) on catecholamine synthesis and TH mRNA levels in PC12 cells. Life Sci. 2000;66: PL303–311. 10834306 10.1016/s0024-3205(00)00549-x

[17] Springer J, Azer J, Hua R, Robbins C, Adamczyk A, McBoyle S, et al The natriuretic peptides BNP and CNP increase heart rate and electrical conduction by stimulating ionic currents in the sinoatrial node and atrial myocardium following activation of guanylyl cyclase-linked natriuretic peptide receptors. J Mol Cell Cardiol. 2012; 52: 1122–1134. 10.1016/j.yjmcc.2012.01.018 22326431

[18] Mosqueda-Garcia R, Furlan R, Tank J, Fernandez-Violante R. The elusive pathophysiology of neurally mediated syncope. Circulation. 2000; 102: 2898–2906. 11104751 10.1161/01.cir.102.23.2898

[19] Mosqueda-Garcia R, Furlan R, Fernandez-Violante R, Desai T, Snell M, Jarai Z, et al Sympathetic and baroreceptor reflex function in neurally mediated syncope evoked by tilt. J Clin Invest. 1997; 99: 2736–2744. 9169504 10.1172/JCI119463 PMC508120

[20] Zhao J, Du S, Yang J, Lin J, Tang C, Du J, et al Usefulness of plasma copeptin as a biomarker to predict the therapeutic effectiveness of metoprolol for postural tachycardia syndrome in children. Am J Cardiol. 2014; 114: 601–605. 10.1016/j.amjcard.2014.05.039 24996552

[21] Freeman R, Wieling W, Axelrod FB, Benditt DG, Benarroch E, Biaggioni I, et al Consensus statement on the definition of orthostatic hypotension, neurally mediated syncope and the postural tachycardia syndrome. Clin Auton Res. 2011; 21:69–72. 10.1007/s10286-011-0119-5 21431947

[22] Winker R, Barth A, Dorner W, Mayr O, Pilger A, Ivancsits S, et al Diagnostic management of orthostatic intolerance in the workplace. Int Arch Occup Environ Health. 2003;76: 143–150. 12733087 10.1007/s00420-002-0395-4

[23] Zhang F, Li X, Ochs T, Chen L, Liao Y, Tang C, et al Midregional pro-adrenomedullin as a predictor for therapeutic response to midodrine hydrochloride in children with postural orthostatic tachycardia syndrome. J Am Coll Cardiol. 2012; 60: 315–320. 10.1016/j.jacc.2012.04.025 22813609

[24] Yang J, Zhao J, Du S, Liu D, Fu C, Li X, et al Postural orthostatic tachycardia syndrome with increased erythrocytic hydrogen sulfide and response to midodrine hydrochloride. J Pediatr. 2013;163: 1169–1173. 10.1016/j.jpeds.2013.04.039 23726544

[25] Low PA, Sandroni P, Joyner M, Shen WK. Postural tachycardia syndrome (POTS). J Cardiovasc Electrophysiol. 2009; 20: 352–358. 10.1111/j.1540-8167.2008.01407.x 19207771 PMC3904426

[26] Winker R. Orthostatic intolerance—prevalence, diagnostic management and its significance for occupational medicine. Wien Klin Wochenschr. 2004;116:40–46. 15518091

[27] Zhang FW, Liao Y, Li XY, Chen L, Jin HF, Du JB. Therapies for postural tachycardia syndrome in children. Zhonghua Er Ke Za Zhi. 2011; 49: 428–432. 21924055

[28] Zhang Q, Chen X, Li J, Du J. Orthostatic plasma norepinephrine level as a predictor for therapeutic response to metoprolol in children with postural tachycardia syndrome. J Transl Med. 2014; 12:249 10.1186/s12967-014-0249-3 25204388 PMC4177336

[29] Jarjour IT. Postural tachycardia syndrome in children and adolescents. Semin Pediatr Neurol. 2013; 20: 18–26. 10.1016/j.spen.2013.01.001 23465770

[30] Liao Y, Yang J, Zhang F, Chen S, Liu X, Zhang Q, et al Flow-mediated vasodilation as a predictor of therapeutic response to midodrine hydrochloride in children with postural orthostatic tachycardia syndrome. Am J Cardiol. 2013; 112: 816–820. 10.1016/j.amjcard.2013.05.008 23735645

